# The Linguistic–Cognitive Profile in an Adult Population with Parkinson’s Disease and Deep Brain Stimulation: A Comparative Study

**DOI:** 10.3390/ejihpe14020026

**Published:** 2024-02-15

**Authors:** Alejandro Cano-Villagrasa, Miguel López-Zamora, Lorena Romero-Moreno, Beatriz Valles-González

**Affiliations:** 1 Faculty of Health Sciences, Valencian International University (VIU), 46002 Valencia, Spain; beatriz.valles@professor.universidadviu.com; 2 Department of Developmental and Educational Psychology, University of Malaga, 29010 Malaga, Spain; miglopzam@uma.es; 3 Department of Neurosurgery, Regional University Hospital of Malaga, 29010 Malaga, Spain; lorena.romero.sspa@juntadeandalucia.es

**Keywords:** Parkinson’s disease, deep brain stimulation, language, cognitive

## Abstract

*Introduction.* Individuals with Parkinson’s disease (PD) exhibit general impairments, particularly non-motor symptoms that are related to language, communication, and cognition processes. People with this disease may undergo a surgical intervention for the placement of a deep brain stimulation device, which improves their motor symptoms. However, this type of intervention leads to a decline in their linguistic and cognitive abilities that becomes increasingly noticeable as the disease progresses. *Objective*. The objective of this research was to compare the performance and linguistic–cognitive profile of individuals with Parkinson’s disease who underwent deep brain stimulation treatment based on the stage of the disease. *Method*. A total of 60 participants who were diagnosed with PD by their reference hospital were selected. These participants were divided into three groups based on the stage of the disease that they were in, forming three groups: a Stage I group (n = 20), a Stage II group (n = 20), and a Stage III group (n = 20). The linguistic–cognitive profile was assessed using the MoCA, ACE-III, and MetAphas tests. The design of this study was established as a quasi-experimental, cross-sectional investigation, and statistical analysis was performed using MANOVA to compare the scores between the study groups. *Results*. The results indicate that individuals in Stage I exhibit better linguistic and cognitive performance compared to the other groups of participants in Stage II and Stage III, with statistically significant differences (*p* < 0.05). *Conclusion.* In conclusion, the progression of PD leads to significant linguistic and cognitive decline in individuals with this disease who have a deep brain stimulation device, greatly limiting the autonomy and quality of life for people with PD.

## 1. Introduction

Parkinson’s disease (PD) was first described in 1817 by James Parkinson, who defined it as a progressive degeneration of the substantia nigra, accompanied by a set of motor features that present early on as a triad: bradykinesia, rigidity, and resting tremor [1]. This definition remains relevant today in describing the symptomatology of PD [2]. While the causes of this disorder remain a topic of debate on and research in PD, the study of the behavioral and cognitive manifestations of Parkinson’s continues to be of the utmost importance. With the aging population in industrialized countries, the prevalence of age-related diseases is increasing, with an estimated rise from 6.9 million diagnosed individuals in 2015 to 14.2 million in 2040 [3]. This situation creates a specific need to understand the manifestations of PD in order to carry out personalized diagnoses and establish treatments that are tailored to the individual beyond the treatment of motor symptoms. This is because the manifestations, in addition to the classic triad, extend to other areas such as sleep, gastrointestinal problems, mental health, cognitive processes, and language [4].

People with Parkinson’s disease (PwPD) commonly exhibit heterogeneous symptoms, resulting from the involvement of different neural systems that, as mentioned before, are not yet fully defined [5] but are responsible for the impairment of various functions that are categorized into two major groups: motor (e.g., tremor and dystonia) and non-motor symptoms, which can manifest from the early stages of the disease. Among the non-motor symptoms, we can mention sleep problems, gastrointestinal issues, and notably, cognitive impairments that have a significant impact on patients’ quality of life [6]. These cognitive impairments include mental health alterations (such as depression and dementia), sensory alterations (such as hyposmia and pain), and problems related to language and communication. All of these manifestations represent the main therapeutic targets in PD [7].

The available treatments for this disorder are varied. The most common ones focus on the treatment of dementia through the use of cholinesterase inhibitors [6,7,8], selective norepinephrine reuptake inhibitors for depression such as atomoxetine [7,8], cognitive training [8], or neurostimulation. Within this latter type of interventions, deep brain stimulation (DBS) is one of the most recent ones. It involves the implantation of a series of electrodes in areas of the brain such as the thalamus, pallidum, or subthalamic nucleus to provide symptomatic relief from the motor manifestations of PD through the generation of electrical impulses. It is important to note that neurosurgery always carries risks, particularly for elderly individuals. However, DBS also presents evident advantages, such as personalized adjustment to each patient’s characteristics, the possibility of readjustment after the surgery, controllability, and, above all, reversibility, as the electrical stimulation can be activated or deactivated by the patient [6,7,8]. While this technique is relatively new, it is currently expanding and being applied to other disorders such as epilepsy or obsessive–compulsive disorders [9]. However, the underlying mechanisms of its functioning and the true extent of its therapeutic potential beyond motor symptoms are still being studied. Therefore, it is crucial to investigate how PwPD who have undergone DBS respond to this type of intervention beyond motor manifestations and explore its effects on other behavioral manifestations of the disorder. In light of the above, the objective of this study is to analyze the cognitive–linguistic profile of a group of PwPD with DBS devices. This objective is of interest because while the benefits of DBS in terms of the motor manifestations of PD have been confirmed, the evidence regarding its effects on the cognitive or linguistic areas is still under development [10,11]. Among the aspects that deserve further in-depth study is how DBS affects patients throughout the etiological course of PD, as this disorder exhibits clear differences between the first stage and the subsequent two stages.

In Stage I, motor symptoms are generally mild, and no significant changes in language are observed. However, some patients may experience slight decreases in speech clarity, reduced vocal modulation, or subtle changes in speech rhythm [12]. As the disease progresses, speech- and language-related issues may become more evident. In Stage II of Parkinson’s disease, motor symptoms intensify, and speech and language problems become more prominent. Difficulties such as dysarthria, which affects speech articulation and clarity, and hypophonia, characterized by a weak and barely audible voice, may arise [13]. Additionally, there may be a decrease in verbal fluency, and handwriting may become smaller (micrographia). In advanced stages of PD (i.e., Stage III), speech and language problems tend to be more pronounced [14]. Speech may become unintelligible, and phenomena such as palilalia (involuntary repetition of words or phrases) and echolalia (repetition of previously heard words or phrases) may occur. There may also be difficulties in finding the right words, implying a reduction in expressive vocabulary. Furthermore, limitations in following complex instructions or maintaining a coherent conversation may be present [4].

On the other hand, existing research supports the notion that PwPD in Stage I have better cognitive competence compared to those in more advanced stages. The findings from various studies have confirmed this observation [15,16]. As the disease progresses, alterations in cognitive processes have been identified in different phases of the disease. In the early stages of PD, cognitive impairments tend to be subtle and may go unnoticed [17]. However, it has been observed that some individuals may experience difficulties in tasks involving sustained attention, processing speed, and working memory. These symptoms can affect the ability to multitask and maintain concentration for extended periods [18]. As the disease advances to the intermediate phase, more pronounced cognitive impairments are likely to occur. Difficulties in memory, particularly in episodic memory and recognition memory, are observed [19]. Additionally, executive function can be affected, manifesting as difficulties in planning, organizing, and problem solving. Difficulties in cognitive processing speed and selective attention may also arise. In the advanced stages of PD, cognitive changes can be more significant, and symptoms of Parkinson’s dementia may develop [20]. Parkinson’s dementia is characterized by significant cognitive decline in areas such as memory, attention, and executive functions [21]. The symptoms can be similar to those observed in Alzheimer’s disease, such as long-term memory loss and spatial and temporal disorientation [15,22,23].

Within the symptomatology of PD, the study of linguistic impairments is often not a priority. This is typically because the most severe symptoms tend to manifest in the advanced stages of the disease or because they may be masked by cognitive and/or mental health issues. However, for authors like Murray [23], language takes on great importance in highlighting the functioning of individuals who are diagnosed with Parkinson’s disease, as the neuroanatomical evidence of language-related alterations in PwPD is becoming increasingly conclusive and significant. For example, it has been discovered that PwPD exhibit a decrease in gray matter in regions that are associated with language, such as the middle frontal gyrus (MFG), the opercular part of the inferior frontal gyrus (IFG), the transverse temporal gyrus (TTG), and the planum temporale (PT).

Linguistic function can be affected in PwPD due to difficulties being encountered in tasks such as finding the right words, forming sentences fluently, and maintaining a normal conversational rhythm. As these impairments worsen, the effectiveness of communication can be compromised in various aspects.

Altmann and Troche [24] assert that PwPD exhibit deficiencies in the production of complex language, such as reduced informational content, deteriorated grammaticality, interrupted fluency, and reduced syntactic complexity. On the other hand, Montemurro et al. [25] suggest that specific aspects of pragmatics, both in production and comprehension, may be affected in PwPD. Yokoi et al. [26] found that PwPD used fewer morphemes in a sentence compared to healthy individuals.

Hochstadt et al. [27] state that a difficulty in understanding sentences in PwPD may be linked to deficits in task switching, verbal working memory, and cognitive flexibility. In another study, Miller et al. [28] found that PwPD exhibited various impairments in their functional communication, resulting in difficulties in following a conversation, expressing opinions or emotions, and contributing to the conversation with relevant topics.

Language disorders can also manifest without an association with dementia, which requires special attention from speech–language pathologists during the initial evaluation. In this regard, Tremblay et al. [29] and Prieto et al. [30] found deficits in understanding metaphors and irony in this population, as well as difficulties in social cognition or Theory of Mind that compromised the quality of exchanges in the context of conversation. In a recent study, Hoz et al. [31] concluded that PwPD may present deficits in lexical-semantic processing, syntactic organization, and fluency.

Tremblay et al. [29] found deficiencies in the comprehension of metaphors and irony in this population, as well as difficulties in social cognition or Theory of Mind, which compromised the quality of exchanges in the context of conversation in PwPD without other more evident cognitive limitations. Cano Villagrasa et al. [32] conducted a descriptive study, aimed at understanding the clinical–epidemiological profile of PD and the coexistence of different types of symptoms. The results concluded that PwPD present symptoms that can be classified as motor symptoms (MSs), non-motor symptoms (NMSs), and speech and language symptoms (SLSs). The latter include limitations in vocal, respiratory, expressive, and receptive language functions, as well as swallowing difficulties (dysphagia). This leads speech–language pathologists to propose the use of different strategies that involve physical, cognitive, and pragmatic efforts, requiring self-awareness or self-perception on the part of the PwPD regarding their symptoms and a conscious use of resources to improve them.

PD causes alterations in front-subcortical neurological functioning, which is why cognitive symptoms can vary widely in terms of type and impact. Some authors indicate that around 80% of PwPD may experience some form of mild cognitive impairment (MCI). Cummings [33] and Bayles and Tomoeda [1] consider that dementia may affect 20–40% of PD cases. PwPD and their caregivers often report that cognitive decline is one of their major concerns. MCI affects approximately 20–50% of people with PD, and longitudinal studies reveal dementia in up to 80% of PD cases [34].

Regarding the causes, neuroimaging studies in PwPD have detected cortical volume loss in the posterior, parietal, and frontal cortices, as well as atrophy in the hippocampus, insula, and cingulate gyrus, which correlates with alterations in cognitive and mnemonic processes [35]. Another aspect related to cognition, such as executive functioning (higher cognitive skills that allow us to set goals, make decisions, regulate our behavior, and solve problems), may be compromised, leading to difficulties in switching between tasks, inhibiting automatic responses, or solving complex problems [34]. These neuroanatomical alterations may be associated with cognitive manifestations that are linked to difficulties in executive function or memory, which have a significant impact on patients [1]. These difficulties have negative effects on attention and task switching, resulting in limitations in engaging in efficient conversations by being unable to follow the flow of the conversation. Aracil-Bolaños et al. [36] corroborate that cognitive impairment is a significant disabling feature in PD, although not all PwPD will experience these symptoms. Some common cognitive symptoms associated with PD include alterations in attention and concentration, memory impairments, slowed cognitive processing, and executive dysfunction.

Thus, communication limitations in PwPD may be related to cognitive impairments such as decreased attention and concentration. PwPD may have difficulties maintaining attention and focusing on specific tasks, which can affect their ability to complete complex tasks or engage in lengthy conversations. Hochstadt et al. [27] state that memory can be affected in PD, especially working memory, which involves the ability to temporarily retain and manipulate information. Difficulties in episodic memory, which refers to the ability to recall specific events and autobiographical details, may also be present. Additionally, PD can slow down information processing, resulting in increased difficulty in thinking quickly, making decisions, and responding efficiently to stimuli [18,19]. Executive dysfunction in PwPD, due to the characteristic neurological alterations of the disease, can have significant repercussions in various aspects of language and communication. A difficulty in planning and organizing may result in problems accessing the right words during conversation, a phenomenon known as “verbal blocking.” Likewise, a lack of cognitive flexibility can contribute to speech rigidity and a reduced ability to adapt to changes in conversation topics. Furthermore, impairment in decision making may influence the ability to express thoughts clearly and coherently. The interplay between executive functions and language underscores the complexity of cognitive and communicative challenges in PD. A comprehensive approach to addressing these areas becomes essential for providing effective interventions and enhancing the quality of life for those affected [37]. Understanding these connections not only enables more precise symptom management but also lays the groundwork for designing therapeutic strategies that holistically address the cognitive and communicative needs of PwPD [28].

PwPD may have difficulties in planning and organizing tasks, which can affect their ability to carry out action sequences or meet deadlines. It is important to note that the severity and extent of cognitive symptoms can vary from person to person and throughout the progression of the disease. Some PwPD may experience mild cognitive symptoms that do not significantly impact their daily lives, while in other cases, the symptoms may be more pronounced and require specific intervention and support. It is expected that when PwPD experience advanced cognitive decline, linguistic symptoms become more prominent, and there are more pronounced limitations in lexical access, sentence structure, or comprehension of complex texts [28,29,30,31].

In PD, language disorders that are associated with executive functioning impairments can be detected. In some cases, linguistic symptoms are associated with mild cognitive impairment or dementia. These alterations can affect the complex processes underlying pragmatic function, as well as sentence comprehension and lexical–semantic processing [38]. According to Chaudhuri and Schapira [18], perceptual impairments, cognitive decline, and affective–behavioral changes (anxiety, depression, apathy) will have a significant impact on communication and language.

Recent research has studied the interrelation between cognitive deficits and communication in PwPD [39,40,41]. Symptoms such as depression and sleep disorders seem to have a negative impact on the linguistic functioning of PwPD, particularly affecting prosody and hypophonia [33]. Therefore, there is a relationship between different groups of symptoms. Additionally, other studies have shown that symptoms like depression can affect the overall functioning of PwPD [42], which can consequently impact the quality of communication.

Clarifying these questions allows for the development of an appropriate intervention plan and the definition of the most suitable courses of action. Unfortunately, speech and language symptoms may manifest even before assessment scores fall outside the normal ranges [43]. Furthermore, their presence can be very subtle and may not be perceived by the patient, such as in the case of dysphagia or cognitive and language impairments. Cardoso and Luchesi [44] propose that individuals with neurodegenerative diseases may perceive changes in communication, social isolation, lack of motivation, and loss of self-esteem. These changes can lead them to withdraw from contact with others, alter their behavior, and avoid society in general or situations that expose them in any way.

Thus, the evaluation process should be seen as complex, dynamic, and continuous. It is complex in the sense that each PwPD will present a unique profile of functioning associated with numerous factors such as the age of onset of PD, effectiveness of medication, access to non-pharmacological therapies, comorbidity with other conditions (depression, respiratory disorders, cardiovascular diseases, among others), and treatment adherence. It is dynamic because the profile of functioning will be modified by the inevitable progression of the disease or health complications, requiring ongoing adjustments in care. And it is continuous because PwPD require comprehensive attention that ensures communicative functionality and the best possible quality of life in the various stages of PD.

Taking this approach into consideration, the main objective of the present study was to compare the cognitive and linguistic profile across stages in three groups of PwPD who have undergone treatment through deep brain stimulation. Specific objectives were also established: (I) to explore difficulties in language and communication skills among the participants, and (II) to observe alterations in cognitive abilities in this population. Finally, two research hypotheses were determined: the first research hypothesis posited that performance in language and communication tasks would be better in PwPD in Stage I compared to Stage II, and in turn, better in Stage II compared to Stage III. On the other hand, the second research hypothesis suggested that PwPD in Stage I would demonstrate better cognitive performance than those in Stages II and Stage III.

## 2. Materials and Methods

### 2.1. Participants

This study included a total of 60 participants (31 males and 29 females), ranging in age from 60 to 79 years (M = 69.4; SD = 8.43), who were diagnosed with PD at different stages and had a deep brain stimulation device. Based on this, three groups were established: a group of participants in Stage I (n = 20), a group of participants in Stage II (n = 20), and a group of participants in Stage III of PD (n = 20). All participants were evaluated at a reference hospital, where they received the diagnosis and underwent rehabilitation processes through the services of physiotherapy, speech therapy, neuropsychology, and occupational therapy. The degree of disability was assessed at the participant’s reference healthcare center through national disability and dependency assessment scales.

This study established inclusion and exclusion criteria that were tailored to different stages of PD. In Stage I, a confirmed diagnosis by a public medical team, active participation in multidisciplinary interventions, the use of a deep brain stimulation device, the ability to communicate clearly in everyday situations, and basic cognitive functions without significant deficits were required. In Stage II, additional criteria included the ability to address potential language difficulties, orientation in time and space, and the presence of non-significant memory loss. In Stage III, an advanced ability to maintain fluid conversations and comprehend instructions was highlighted, with minimal competence in cognitive tasks such as planning and decision making. Exclusion criteria encompassed the presence of severe sensory impairment, intellectual disability limiting participation, residence in a nursing home, and current employment. These criteria were designed to ensure homogeneity within the groups, allowing for an equitable representation of the specific characteristics of each stage of PD and facilitating a precise analysis of linguistic and cognitive variables in the study. Furthermore, individuals with comorbidities of neurological or neurodegenerative damage along with Parkinson’s disease were excluded.

With these criteria in mind, the sample selection was carried out through an initial survey in which a total of 173 participants were collected. Out of the total, 63 were excluded for not meeting the established inclusion and exclusion criteria. From the remaining 110 participants, 27 were further excluded due to a lack of technological means to perform the assessments, and 23 participants did not provide signed informed consent. Thus, the three experimental groups were formed.

Table 1 presents the main sociodemographic characteristics of the participants included in the study.

### 2.2. Instruments

**Montreal Cognitive Assessment (MoCA)** [44]. The MoCA test is a cognitive screening tool that is used to assess the cognitive profile of individuals suspected of cognitive impairment. This test consists of eight tasks that evaluate orientation, short-term memory, executive function/visuospatial abilities, language skills, abstraction, animal naming, attention, and clock drawing test. The maximum score is 30. Its psychometric characteristics describe a high level of reliability and validity, with a sensitivity of 87% and a specificity ranging from 90% for mild cognitive impairment (MCI), using a cutoff score of <26, and a sensitivity of 87% for Alzheimer’s dementia with a specificity of 100%, using a cutoff score of <18. In this test, only the variables of naming, abstraction, deferred recall, and orientation were selected.

**Addenbrooke’s Cognitive Examination III (ACE-III)** [45]. The ACE-III consists of 21 questions, with a total score of 100. The maximum score for the language area is 26 points. It assesses six cognitive domains, with a maximum score of 100 points: orientation (10 items), attention (8 items), memory (35 items), verbal fluency (14 items), language (28 items), and visuospatial abilities (5 items). One point is assigned for each correct response. According to the authors of the Spanish version, the sensitivity and specificity of the exam are reported as 90% and 86%, respectively, using a cutoff score of 68 points. The thresholds describe the score at which a diagnosis of cognitive impairment should be considered and are usually 82 or 88/100. For this research, only the subtests of fluency, language, attention, memory, and visuospatial abilities were used.

**MetAphAs Test** [46]. Through the application of this test, it is possible to define the metalinguistic performance of individuals with cognitive impairment and identify differential metalinguistic profiles, which serve as a starting point for planning individualized interventions. This test includes 40 items distributed across six sections as follows: (1) Inner language, inhibition ability, and discourse. (2) Simultaneous control of semiotic procedures. (3) Paraphrasing skills and associated phenomena. (4) Reported speech and associated phenomena. (5) Monitoring ability. Contextualization marks. (6) Use of displaced language and Theory of Mind (ToM). For this research, only the final section (Section 5) was recorded, which helps assess fundamental skills for information exchange related to describing an absent (or hidden) object or situation, temporal displacement I (recent past), temporal displacement II (remote past), temporal displacement III (near future), interpreting a scene, searching for antonyms, reading emotions, using fictional language, ability to lie, and capacity to understand irony. Section 6 is used to evaluate different functions related to Theory of Mind. All functions are evaluated with a score ranging from 0 to 4, with a score of 0 to 2 considered an indicator of poor performance.

### 2.3. Procedure

This study was approved by the ethics committee of the Universidad Católica de Murcia (UCAM), with the code CE052206. The data collection process involved evaluating the participants in a single session lasting 1 h and 30 min. The language assessment tests consisted of administering tasks related to naming skills, verbal fluency, and lexical access. Likewise, the tests for cognitive evaluation involved word detection, memorization of elements, and performing mathematical calculations. The participants were accompanied throughout the process by a family member or primary caregiver. The data from the measurement instruments were stored in protected databases, which were subsequently analyzed by the research team members to verify the fulfillment of the research hypotheses.

### 2.4. Design

In this quasi-experimental, cross-sectional study, various statistical analyses were conducted. First, the Shapiro–Wilk test was used to confirm the assumption of normality for the dependent variables that make up the study groups. Next, Multivariate Analysis of Variance (MANOVA) was used to examine the relationship between the variables related to the participants’ cognitive and linguistic profile. In this way, differences in the mean scores among the three groups that make up the sample were analyzed. Subsequently, Analysis of Covariance (ANCOVA) tests were conducted to observe the individual differences of each variable in the three groups. In this study, only the variables from the assessment instruments that corresponded to the cognitive and linguistic sections were selected. Variables that did not record these types of skills were discarded, as well as any repeated variables in the instruments, with a summation performed among the obtained direct scores. Finally, to control for Type I error, Holm–Bonferroni correction was applied.

## 3. Results

### 3.1. Differences in the Linguistic Profile among the Groups

**Figure 1:**
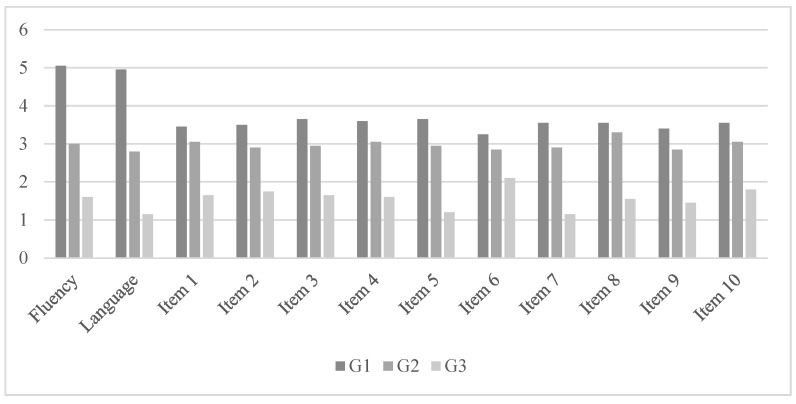
Mean scores of measures related to language skills.

The ANCOVA that was conducted to assess differences in linguistic profile measures among Stage 1 (G1), Stage 2 (G2), and Stage 3 (G3) revealed the presence of statistically significant differences (Wilks’ Lambda = 0.028, F(6,52) = 19.016, *p* < 0.001, η^2^_P_ = 0.832). As shown in Table 2, the variables in which significant differences were found were fluency, language, Item 1, Item 2, Item 3, Item 4, Item 5, Item 6, Item 7, Item 8, Item 9, and Item 10. The results of the ANCOVAs related to the linguistic profile are presented in Table 2 and Figure 1.

The post hoc analysis indicates that the group of participants diagnosed with PD at Stage I performed significantly better in linguistic scores compared to the group at Stage II and the group at Stage III (*p* < 0.05). The group at Stage II also showed significantly better scores compared to the group at Stage III.

### 3.2. Differences in the Cognitive Profile among the Groups

**Figure 2:**
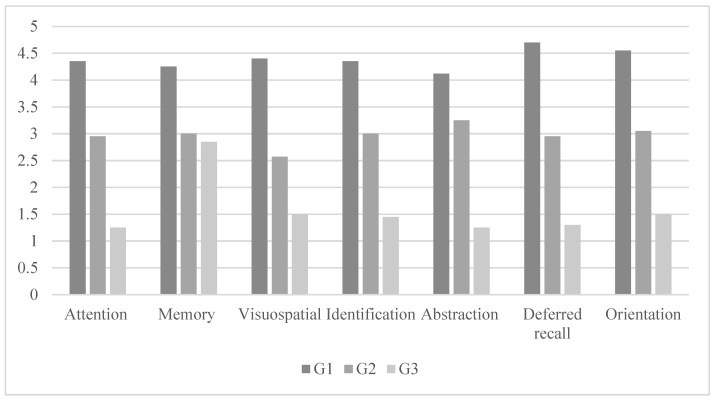
Mean scores of measures related to cognitive skills.

The ANCOVA that was conducted to assess differences in cognitive profile measures among Stage 1 (G1), Stage 2 (G2), and Stage 3 (G3) revealed statistically significant differences (Wilks’ Lambda = 0.036, F(6,52) = 31.034, *p* < 0.001, η^2^_P_ = 0.810). As shown in Table 3, the variables that yielded significant differences were attention, memory, visuospatial abilities, identification, abstraction, delayed recall, and orientation. The results of the ANCOVAs related to the cognitive profile are presented in Table 3 and Figure 2.

The post hoc analysis indicates that the group of participants diagnosed with PD in Stage I showed higher cognitive performance compared to the group in Stage II, followed by the group in Stage III, with statistically significant differences (*p* < 0.05).

## 4. Discussion

As previously stated, the main objective of this study was to analyze the cognitive–linguistic profile and compare it based on the stage of PD in which individuals were diagnosed. Accordingly, a series of specific objectives were selected, which included exploring the difficulties in language and communication skills of participants who were diagnosed with PD, as well as observing the alterations in cognitive abilities within this population.

The results of our study confirm the first research hypothesis, which stated that the performance of language tasks would be better in participants who were diagnosed with PD in the early stages (Stage I) compared to those in the later stages (Stages II and III). In the present study, participants in Stage I of the disease showed higher scores on language tasks compared to those in Stage II, followed by those in Stage III. These findings indicate that the linguistic profile of PwPD is better preserved in Stage I than in more advanced stages of the disease. In this stage, motor symptoms are typically mild, and significant changes in language are not observed. However, some patients may experience slight decreases in speech clarity, reduced vocal modulation, or subtle changes in speech rhythm. As the disease progresses, speech and language problems may become more evident. Common linguistic symptoms in this stage include dysarthria, hypophonia, decreased verbal fluency, and micrographia. Finally, in the advanced stages of PD, speech and language problems are typically more pronounced. Common linguistic changes include unintelligible speech, palilalia, echolalia, as well as decreased vocabulary and difficulty finding the right words, along with limitations in following complex instructions or maintaining coherent conversation [47].

These findings align with results obtained in studies such as that by Bocanegra et al. [48], who used different language tests and assessments. The researchers found significant differences in linguistic impairment between patients with idiopathic PD and patients with genetic parkinsonism. These differences were observed in areas such as speech fluency, verbal production, and language comprehension. Similarly, in the study by Nishiwaki et al. [49], the researchers used a computerized semantic association test to evaluate participants’ language skills. This test involved presenting a target word and asking participants to generate associated words within a specific time frame. Both the quantity and quality of the provided responses were analyzed. The results revealed that PwPD showed significant impairments in the evaluated language skills compared to a control group. PwPD had difficulties generating appropriate responses and exhibited reduced verbal fluency and word variety.

Regarding the second research hypothesis, which posited that PwPD in Stage I would exhibit better cognitive competence than those in more advanced stages, the results obtained in this study confirm it. PwPD experience cognitive changes as they progress through the stages of the disease. In the early stages of PD, cognitive alterations are often subtle and may go unnoticed. However, it has been observed that some individuals may have difficulties in tasks that require sustained attention, processing speed, and working memory. These symptoms can affect the ability to multitask and maintain focus over prolonged periods [19]. As PD advances to the intermediate phase, more pronounced cognitive impairments are likely to occur. Difficulties in memory, particularly in episodic memory and recognition memory, may become more evident [20]. Additionally, executive function may be affected, resulting in difficulties in planning, organizing, and problem solving. There may also be difficulties in cognitive processing speed and selective attention. Lastly, in the advanced stages of PD, cognitive changes can be more significant and may include the onset of Parkinson’s dementia [21]. The dementia in PD is characterized by significant cognitive impairment in areas such as memory, attention, and executive functions. The symptoms can be similar to those observed in Alzheimer’s disease, such as long-term memory loss and spatial and temporal disorientation [24]. The results obtained in this study are consistent with other research, such as that by Pedersen et al. [49], whose findings revealed that approximately half of the participants showed cognitive stability during the study period, without significant deterioration in the evaluated cognitive functions. Around one-third of the participants experienced significant cognitive decline, indicating progression from mild cognitive impairment to dementia. Additionally, a small percentage of participants showed an improvement in cognitive functions, surpassing the criteria for a mild cognitive impairment diagnosis at baseline.

The information provided above regarding linguistic and cognitive impairments in PD at different stages is relevant to DBS in PwPD. In the early stages of PD, cognitive alterations are often subtle and may go unnoticed. However, as the disease progresses to more advanced stages, difficulties in memory, executive function, and cognitive processing speed can become more pronounced. Additionally, symptoms of dementia in PD, which involve significant cognitive decline, may emerge. When it comes to DBS therapy, both positive and negative effects on the linguistic and cognitive abilities of PwPD have been observed. Some studies have reported improvements in speech fluency and language comprehension in patients treated with DBS, which may be related to the reduction in motor symptoms. However, adverse effects have also been observed in some individuals, such as difficulties in speech fluency, changes in voice, and alterations in memory and attention. These findings suggest that DBS therapy can influence linguistic and cognitive abilities variably in each individual. The response to DBS can be unpredictable and depends on factors such as the precise location of the electrodes and the stimulation of adjacent areas. Therefore, it is essential to conduct a comprehensive assessment and closely monitor to identify any changes in these abilities and adjust the treatment accordingly.

## 5. Conclusions

In summary, PwPD experience language impairments such as problems with verbal fluency, lexical access, and deterioration in the content and form of conversation, as well as cognitive difficulties including memory, attention, and executive function deficits. These impairments worsen as the disease progresses through its stages. All of these factors significantly impact their quality of life and interfere with the proper performance of basic daily activities.

This study had a main limitation regarding the small number of participants that were selected. The characteristics of the sample chosen for this research were quite limited and difficult to access. Although the aim of our study was solely to compare the stages of PD in participants who had undergone surgery for DBS electrode placement, future studies should expand the sample and compare this same group with other participants with PD who do not use DBS.

The implementation of early diagnostic tests in neurodegenerative diseases, such as Parkinson’s, becomes crucial in the context of DBS. Advanced cognitive tools, such as ACE-III, MoCA, and MetAphAs, not only enable a comprehensive evaluation of different cognitive domains but also allow for the detection of anomalous patterns that may indicate cognitive decline in preclinical stages. In Parkinson’s patients undergoing DBS, a dual dynamic is evident: while deep brain stimulation has been proven to be effective in controlling motor symptoms, it has also been observed that, in some cases, it may impact cognitive functions. Early identification of cognitive changes through specialized tests becomes critical for adjusting intervention and rehabilitation strategies. The key aspect lies in the fact that early diagnosis not only enables the timely application of interventions and rehabilitation, improving the patient’s quality of life, but that it may also play a role in slowing down the progression of cognitive impairments. Early intervention encompasses everything from cognitive therapies to environmental adaptations and stress management strategies. In the realm of DBS for Parkinson’s, a comprehensive evaluation that includes cognitive tests, clinical information, medical history, and periodic neurological assessments is essential. The interpretation of these results should be entrusted to specialized healthcare professionals who can design personalized intervention plans and closely monitor the patient’s progress. The combination of early diagnostic tests with comprehensive care and early intervention strategies is essential to optimize the management of cognitive complications in Parkinson’s patients, especially those who have undergone DBS. This approach not only positively impacts the patients’ quality of life but also provides the opportunity to maximize the benefits of deep brain stimulation.

Therefore, this research serves as a starting point to further explore language and cognitive impairments in PwPD. Consequently, there is a need to continue working on research lines that shed light on the linguistic and cognitive characteristics of this population, with the goal of determining their limitations and developing optimal assessment and intervention protocols for individuals with this condition. Additionally, it is essential to propose investigations that determine the benefits and drawbacks for PwPD who have undergone surgical intervention for DBS, in order to establish the degree to which success in the proper performance of basic daily activities can be guaranteed.

## Figures and Tables

**Table 1:** Sociodemographic and individual characteristics of study participants.

		n	Percentage
**Sex**	*Men*	31	51.6
	*Women*	29	48.4
**Diagnosis**	*Stage I*	20	33.3
	*Stage II*	20	33.4
	*Stage III*	20	33.3
**Years of treatment**	*0–5 years*	13	21.7
	*5–10 years*	34	56.6
	*10–15 years*	13	21.7
**Grade of incapacity**	*Less than 33%*	0	0
	*Between 33% and 66%*	47	78.3
	*More than 66%*	13	21.7
**Medication**	*Levodopa*	52	86.7
	*Dopamine agonist*	4	6.7
	*MAO-B enzyme inhibitors*	2	3.3
	*Catechol-O-methyltransferase inhibitors*	2	3.3
**Years since diagnosis**	*14–18 years*	21	35
	*9–13 years*	19	31.7
	*4–8 years*	18	30
	*1–3 years*	2	3.3
**Carer**	*No*	0	0
	*Yes*	60	100

**Table 2:** Differences among the measures of the groups—Stage I (G1), Stage II (G2), and Stage III (G3)—in the linguistic profile obtained from the ACE III test and Section 6 of the MetAphas test.

Profile Linguistic	G1 (n = 20)		G2 (n = 20)		G3 (n = 20)		F _(6,52)_	η^2^_P_	Differences between Groups
	M	SD	M	SD	M	SD			
**ACE—III**									
Fluency	5.05	0.75	3.00	0.79	1.60	0.94	86.34 *	0.75	G3 < G2 < G1
Language	4.95	0.68	2.80	0.69	1.15	1.13	96.93 *	0.77	G3 < G2 < G1
**Section 6 of the MetAphAs test**									
Item 1	3.45	0.51	3.05	0.94	1.65	1.30	18.70 *	0.39	G3 < G2 < G1
Item 2	3.50	0.51	2.90	0.85	1.75	1.20	19.36 *	0.40	G3 < G2 < G1
Item 3	3.65	0.48	2.95	0.75	1.65	1.13	29.31 *	0.50	G3 < G2 < G1
Item 4	3.60	0.50	3.05	0.88	1.60	1.09	28.60 *	0.50	G3 < G2 < G1
Item 5	3.65	0.48	2.95	0.88	1.20	1.10	42.51 *	0.59	G3 < G2 < G1
Item 6	3.25	0.44	2.85	0.87	2.10	1.02	10.19 *	0.26	G3 < G2 < G1
Item 7	3.55	0.51	2.90	0.85	1.15	1.08	42.53 *	0.59	G3 < G2 < G1
Item 8	3.55	0.78	3.30	0.80	1.55	1.14	32.15 *	0.53	G3 < G2 < G1
Item 9	3.40	0.50	2.85	0.74	1.45	0.88	38.03 *	0.57	G3 < G2 < G1
Item 10	3.55	0.51	3.05	0.82	1.80	1.10	22.53 *	0.44	G3 < G2 < G1

**Note.** G1 = Stage I; G2 = Stage II; G3 = Stage III. * *p* < 0.05.

**Table 3:** Differences among the groups—Stage I (G1), Stage II (G2), and Stage III (G3)—in the cognitive profile obtained from ACE-III and MoCA tests.

Profile Cognitive	G1 (n = 20)		G2 (n = 20)		G3 (n = 20)		F _(6,52)_	η^2^_P_	Differences between Groups
	M	SD	M	SD	M	SD			
Attention	4.35	0.48	2.95	0.82	1.25	1.07	69.99 *	0.711	G3 < G2 < G1
Memory	4.25	0.44	3.00	0.91	2.85	0.74	50.55 *	0.639	G3 < G2 < G1
Visuospatial	4.40	0.50	2.57	1.09	1.50	1.10	50.54 *	0.639	G3 < G2 < G1
Identification	4.35	0.48	3.00	0.85	1.45	1.23	61.14 *	0.682	G3 < G2 < G1
Abstraction	4.12	0.43	3.25	0.78	1.25	1.25	79.69 *	0.737	G3 < G2 < G1
Deferred recall	4.70	0.47	2.95	0.75	1.30	1.17	61.80 *	0.684	G3 < G2 < G1
Orientation	4.55	0.51	3.05	0.82	1.50	1.14	55.97 *	0.663	G3 < G2 < G1

**Note.** G1 = Stage I; G2 = Stage II; G3 = Stage III. * *p* < 0.05.

## Data Availability

Participant data is not available due to the data protection and privacy policies carried out in the development of this study.
